# Meniscal centralization significantly improve clinical outcomes and reduce meniscal extrusion with minimal complications: A systematic review

**DOI:** 10.1002/jeo2.70308

**Published:** 2025-06-15

**Authors:** Alessandro Carrozzo, Francesco Bosco, Leandro Ramazzini, Fortunato Giustra, Virginia Masoni, Marcello Capella, Michele Malavolta, Jae‐Sung An, Hideyuki Koga

**Affiliations:** ^1^ Università degli Studi “Link Campus University”, Dipartimento di Scienze della Vita, della Salute e delle Professioni Sanitarie Rome Italy; ^2^ Department of Precision Medicine in Medical, Surgical and Critical Care (Me.Pre.C.C.) University of Palermo Palermo Italy; ^3^ Department of Orthopaedics and Traumatology G.F. Ingrassia Hospital Unit (ASP 6) Palermo Italy; ^4^ Department of Orthopedics Casa di Cura Solatrix Rovereto Italy; ^5^ Department of Orthopaedics and Traumatology Ospedale San Giovanni Bosco di Torino – ASL Città di Torino Turin Italy; ^6^ Department of Orthopaedics and Traumatology University of Turin, CTO Turin Italy; ^7^ Department of Joint Surgery and Sports Medicine, Graduate School of Medical and Dental Sciences Institute of Science Tokyo Japan

**Keywords:** arthroscopy, biomechanics, centralization, knee, meniscus

## Abstract

**Purpose:**

Meniscal extrusion alters joint biomechanics and accelerates cartilage degeneration, contributing to the progression of knee osteoarthritis (OA). Meniscal centralization techniques aim to reposition the meniscus, addressing extrusion and restoring load distribution. This systematic review aims to evaluate meniscal centralization's clinical and radiological outcomes, hypothesizing its efficacy in treating symptomatic meniscal extrusion with minimal complications.

**Methods:**

This review followed Preferred Reporting Items for Systematic Reviews and Meta‐Analyses guidelines and was registered with PROSPERO (CRD42023484353). Literature searches were conducted in PubMed, Science Direct and Scopus. Studies reporting clinical and/or radiological outcomes of meniscal centralization with ≥24 months of follow‐up were included. Data on demographics, surgical techniques, patient‐reported outcome measures (PROMs), imaging findings and complications were extracted. Methodological quality was assessed using the ROBINS‐I tool, and heterogeneity was evaluated via the *I* ^2^ statistic.

**Results:**

Four studies (113 patients, mean follow‐up: 24–35 months) met inclusion criteria. Arthroscopic meniscal centralization with suture anchors significantly improved PROMs, including International Knee Documentation Committee (IKDC), Lysholm and Knee Injury and Osteoarthritis Outcome Score (KOOS) scores, demonstrating symptom relief and functional recovery. Lysholm scores improved from 46.0 to 96.5, KOOS pain from 47.4 to 88.9, and IKDC from 51.8 to 75.8 (*p* < 0.05 for all). Imaging showed reduced meniscal extrusion and improved joint space width. Complications were minimal, though one study reported a 26.9% failure rate due to incomplete healing and OA progression. Rehabilitation protocols allowed return to full activity within 4–6 months.

**Conclusions:**

Meniscal centralization effectively reduces extrusion, improves clinical outcomes, and restores knee function with minimal complications. However, further long‐term and comparative studies are needed to validate these findings and refine surgical indications.

**Level of Evidence:**

Level IV.

**Abbreviations**
ACL: anterior cruciate ligament
BMI: body mass index
BPTB: bone‐patellar tendon‐bone
DFO: distal femoral osteotomy
HKA: hip–knee–ankle
HTO: high tibial osteotomy
IKDC: International Knee Documentation Committee
JLCA: joint line convergence angle
JSW: joint space width
KL: Kellgren–Lawrence
KOOS: knee injury and osteoarthritis outcome score
LM: lateral meniscus
MM: medial meniscus
MME: medial meniscus extrusion
MMPRT: medial meniscus posterior root tear
MRI: magnetic resonance imaging
MT: meniscotibial
OA: osteoarthritis
OWHTO: open‐wedge high tibial osteotomy
PROM: patient‐reported outcome measure
ROBINS‐I: risk of bias in non‐randomized studies of interventions
ROM: range of motion
SIFK: subchondral insufficiency fractures of the knee

## INTRODUCTION

Meniscal extrusion, defined as the meniscus displacement beyond the tibial border, is a critical factor in the progression of knee osteoarthritis (OA) due to its impact on knee joint biomechanics and load distribution [1, 12]. The meniscus plays a critical role in load distribution in the knee joint, and its extrusion disrupts this balance, leading to increased stress on the articular cartilage, which accelerates cartilage degeneration and the development of OA [3, 6, 14].

Meniscal extrusion and posterior root tears are closely related and often lead to rapid joint degeneration. Recent studies have highlighted the importance of the meniscotibial (MT) ligament in stabilizing the medial meniscus (MM) and preventing extrusion [4, 10, 16, 20]. Disruption of the MT ligament has been identified as an early event preceding medial meniscal posterior root tears (MMPRTs), suggesting a progressive pathological sequence in which MT ligament damage leads to meniscal extrusion and subsequent root tears [11].

Surgical techniques such as meniscal centralization have been developed to reposition the meniscus and restore its load‐bearing function. The meniscal centralization, generally performed using suture anchors secured to the tibial plateau to fix the meniscus in place, aims to restore normal meniscal positioning and function, addressing both meniscal extrusion and underlying ligamentous insufficiency to prevent further joint damage [13, 15, 18, 20].

Different procedures have shown promise in effectively reducing meniscal extrusion and leading to good clinical outcomes, both in cases of isolated meniscal extrusion and those involving medial meniscus posterior root tears (MMPRTs) [9, 17]. However, there is a paucity of clinical outcome data on meniscal centralization, particularly regarding its long‐term efficacy and the role of associated procedures in preventing OA progression. A review of the current evidence was therefore warranted.

The objective of this systematic review was to synthesize the current evidence regarding meniscal extrusion and centralization, reporting the clinical outcomes associated with these surgical procedures.

The hypothesis was that these procedures would be effective in treating symptomatic meniscal extrusion, with a low failure rate and minimal complications.

## METHODS

### Literature search

This systematic review was conducted according to the Preferred Reporting Items for Systematic Reviews and Meta‐Analyses guidelines. The study protocol was registered with PROSPERO (registration number: CRD42023484353). A comprehensive literature search was performed in May 2024 using PubMed, Science Direct and Scopus databases. Key terms were: (‘medial meniscus’ OR ‘meniscus’ OR ‘meniscal’) AND (‘centralization’ OR ‘centralization’).

### Inclusion and exclusion criteria

Studies were included if they reported clinical and/or radiological outcomes of meniscal extrusion and centralization. The studies were excluded if they were non‐original research articles, in vitro studies, used animal models, did not report explicit and quantitative data on clinical outcomes, had a follow‐up period of less than 12 months or were not published in English.

### Data extraction and quality assessment

Two reviewers independently (A.C. and F.B.) screened titles and abstracts of identified studies for eligibility. Full texts of potentially relevant articles were retrieved and assessed for final inclusion. Disagreements were resolved by consensus or by a third reviewer (F.G.).

Data extraction was performed using a predesigned data collection form that included study design, patient demographics, details of surgical techniques used, clinical outcomes (including patient‐reported outcome measures [PROMs]), imaging outcomes (such as reduction in meniscal extrusion on magnetic resonance imaging [MRI] or ultrasound), and complication rates and types.

**Figure 1:**
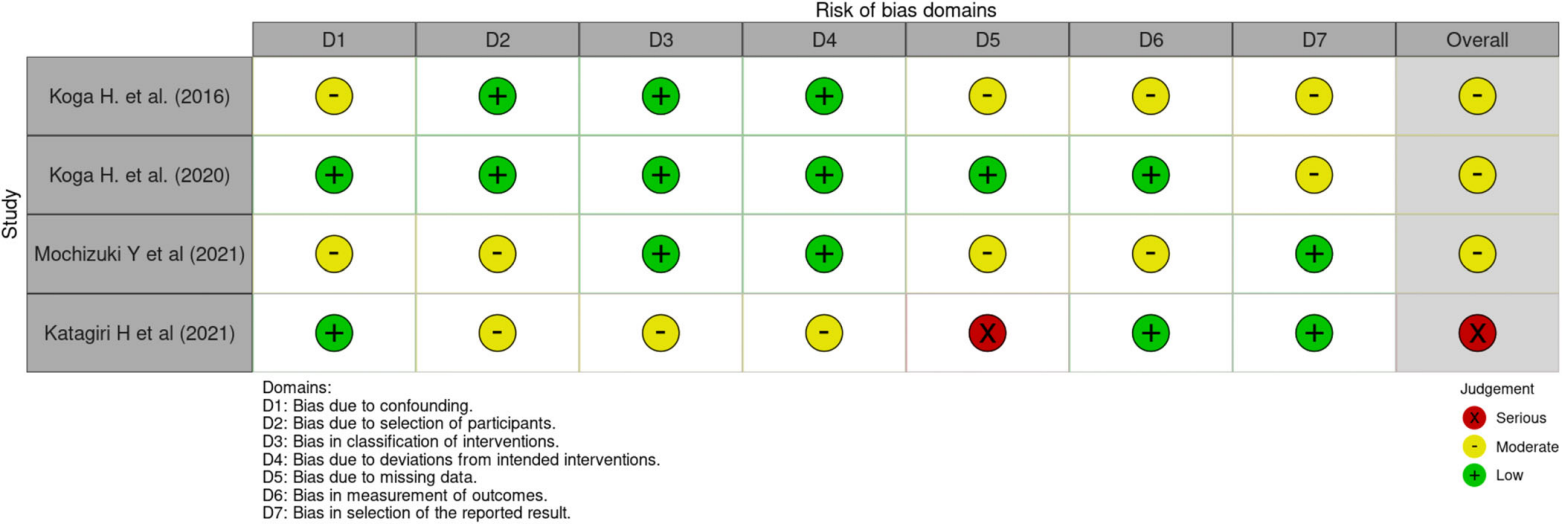
Assessment of the methodological quality of the included studies according to the ROBINS‐I tool. ROBINS‐I, Risk Of Bias In Non‐randomized Studies of Interventions.

The methodological quality of the included studies was assessed using the Risk Of Bias In Non‐randomized Studies of Interventions (ROBINS‐I) tool (Figure 1).

### Statistical analysis

Descriptive statistics were used to summarize the data. Continuous variables were reported as means with standard deviations or medians with interquartile ranges, depending on the distribution of the data. Categorical variables were expressed as frequencies and percentages. Heterogeneity was assessed using the *I* ^2^ statistic, with values greater than 50% indicating substantial heterogeneity.

All analyses were performed with SPSS® Statistics software (version 28.0.0.1; IBM SPSS).

## RESULTS

### Studies characteristics

**Figure 2:**
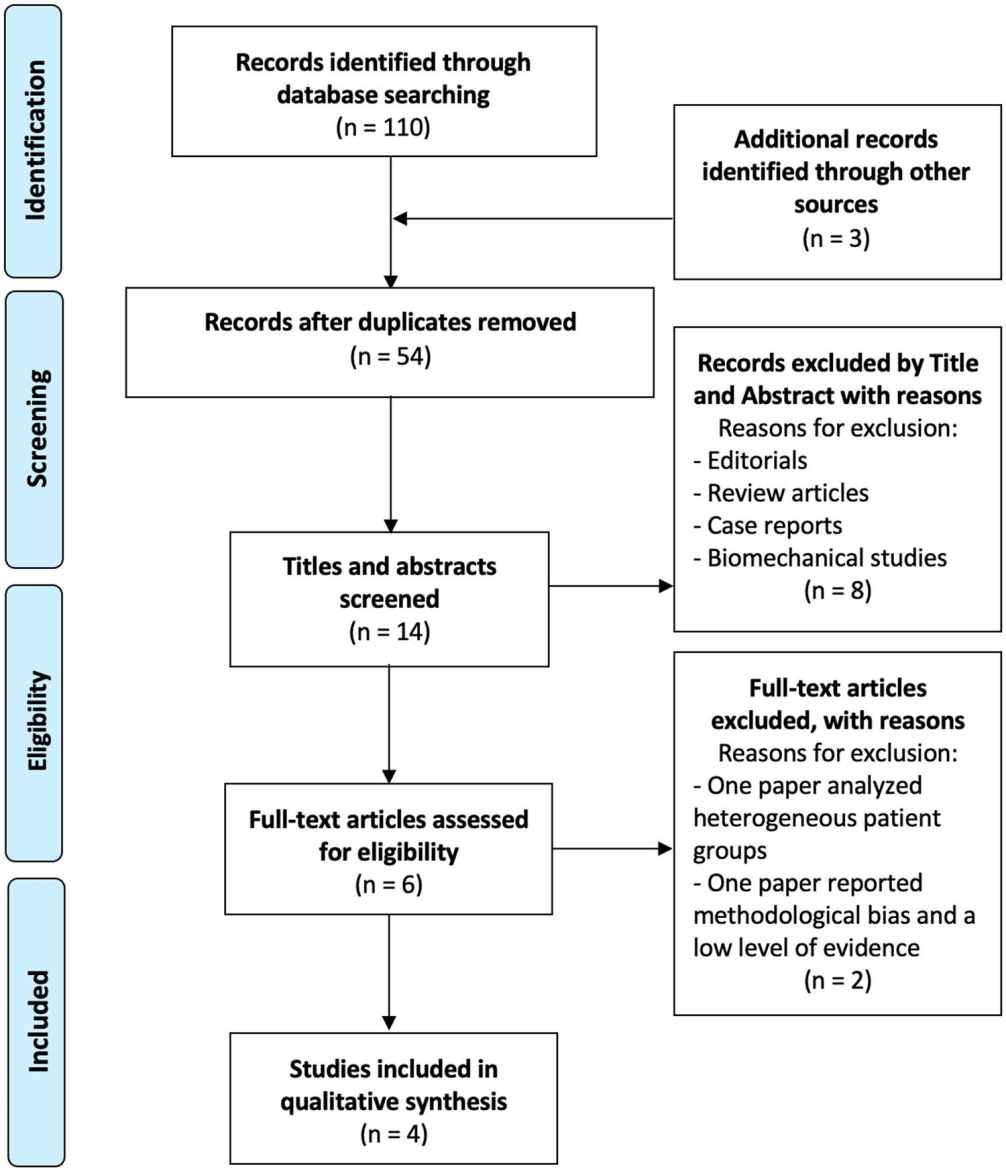
The PRISMA flow diagram outlines the study selection process. PRISMA, Preferred Reporting Items for Systematic Reviews and Meta‐Analyses.

After an initial selection of 110 articles and the removal of duplicates, 54 studies were further analyzed. After applying inclusion and exclusion criteria, four studies focusing on meniscal extrusion and centralization outcomes were included in this systematic review (Figure 2). These studies ranged from 2016 to 2021, and the included studies were case series (level of evidence, 4).

The primary indications for surgery were symptomatic knee pain with meniscal extrusion, after failure of conservative treatment. Inclusion criteria varied but generally required meniscal extrusion of at least 3 mm, as confirmed by MRI, eventually in combination with an MMHPRT. In contrast, exclusion criteria included factors such as malalignment or severe OA. Details on the included studies are reported in Table 1.

**Table 1:** Study details.

Authors and publication year	Journal	Study design	LoE	Inclusion criteria	Exclusion criteria	Indications for surgery	Patients sample size; initial cohort/final cohort, *N*	Patients lost follow‐up or not completing the study, *N*	Follow‐up time (months)
Koga et al., 2016 [7]	Arthroscopy: The Journal of Arthroscopic and Related Surgery	Case Series	IV	LM arthroscopic centralization regardless of history of injuries or surgery in the ipsilateral knee	History of symptomatic injuries in the contralateral knee	GA: Symptomatic knee after conservative treatment, with OA KL 0–2 at lateral compartment or after LM partial meniscectomy, with extrusion of midbody of the LM confirmed by a coronal MRI (≥3 mm)^a^	26/21 (GA: 9 at the analyses of post‐operative results one further patient excluded; GB: 12)	5 (lost within 2 years of follow‐up)	24
						GB: Symptomatic torn discoid meniscus after conservative treatment			
Koga et al., 2020 [8]	The American Journal of Sports Medicine	Case Series	IV	Meniscoplasty by capsular advancement for lateral OA attributed to LM defects regardless of history of injuries or surgery in the ipsilateral knee	Valgus alignment	Symptomatic knee with neutral alignment (mechanical axis <60%) after conservative treatment; OA Grades 3–4 KL at the lateral compartment attributed to LM defects, regardless of history of meniscectomy; age too low for arthroplasty and/or high level of activity and desire to continue sports activity^a^	29/27	2 (lost within 2 years of follow‐up)	24
Mochizuki et al., 2021 [16]	European Journal of Orthopaedic Surgery & Traumatology	Case Series	IV	MM extrusion more than 3 mm on MRI caused by MMPRT; arthroscopic centralization as an augmentation of MMPRT repair; no joint contracture; no history of ipsilateral knee joint surgery; minimum 2 years of follow‐up^a^	OA KL 3–4; varus knee with HKA angle ≥0°; LM extrusion^a^	/	– 47 (Patients with meniscus extrusion who underwent repair) – 26 included in the final analysis – 21 Patients excluded due to exclusion criteria	/	35.4 ± 12.9 (26–60)^b^
Katagiri et al., 2021 [5]	Journal of Knee Surgery	Case Series	IV	OWHTO of physically active patient with unicompartimental knee OA: G1 centralization group (when MM margins extension of at least 3 mm beyond the tibial margin on coronal MRI) G2 control group	1. Patients who refused to provide consent; 2. Patients who joined a stem cell clinical trial; 3. Incomplete questionnaire response; 4. Loss to follow‐up within 2 years.	/	– 50 patients in G1 + G2 – 39 included for the analysis (G1: 21; G2: 18) – 11 Patients excluded due to exclusion criteria	3 (lost within 2 years of follow‐up, already excluded following exclusion criteria)	At least 24 months as inclusion criteria

Abbreviations: HKA, hip–knee–ankle angles; KL, Kellgren–Lawrence; LM, lateral meniscus; LoE, level of evidence; MM, medial meniscus; mm, millimetres; MMPRT, medial meniscus posterior root tear; MRI, magnetic resonance imaging; N, number; OA, osteoarthritis; OWHTO, open‐wedge high tibial osteotomy; SD, standard deviation; /, not reported/not mentioned in the paper.

^a^ All features should be present as criteria. GA: Extrusion group with extruded LM of 3 mm or more; GB: Discoid group, with primary surgery for discoid LM + centralization. G1 Centralization group: OWHTO + MM centralization; G2 Control group: only OWHTO.

^b^ Values expressed as mean ± SD (range).

### Patient demographics

A total of 113 patients were included, with mean follow‐up periods varying between 24 and 35.4 months.

The mean age of patients across studies varied from 29 to 62 years, with the majority of the sample being females (58.4%). Specifically, the gender distribution across the studies ranged from 47.6% to 69.2% female. Body mass index (BMI) was reported in three studies, with mean values ranging from 23.4 to 24.2 kg/m^2^. Previous surgeries varied, including partial meniscectomies and anterior cruciate ligament (ACL) reconstruction. Patient demographics are reported in Table 2.

**Table 2:** Patient demographics.

Authors and publication year	Age (years old)	Sex, *N* (%)		BMI	Previous surgery, *N* and types
	Mean ± SD (range) or average (range)	*M*	*F*	Mean ± SD (range) or average (range)	
Koga et al., 2016 [7]	29.0 (13–53)^a^	11 (52.4)^a^	10 (47.6)^a^	/	GA: 2 partial lateral meniscectomy (1 simultaneous revision ACL reconstruction with autologous BPTB graft)
Koga et al., 2020 [8]	43.0 (14–58)	12 (44.5)	15 (55.5)	23.4 (16.7–34.1)	20 subtotal lateral meniscectomy (13 normal LM; 7 discoid LM)
Mochizuki et al., 2021 [16]	62.1 ± 6.0 (48–71)	8 (30.8)	18 (69.2)	24.2 ± 4.0 (18.9–35.4)	/
Katagiri et al. 2021 [5]	G1: 57^b^ (44–73)	G1: 9 (42.9)^c^	G1:12 (57.1)^c^	G1: 23.5^b^ (19.1–28.5)	/
	G2: 62^b^ (47–71)	G2: 7 (38.9)^c^	G2:11 (61.1)^c^	G2: 24.2^b^ (18.5–36.7)	

*Note*: GA: Extrusion group with extruded LM of 3 mm or more; GB: Discoid group, with primary surgery for discoid LM + centralization. G1 Centralization group: OWHTO + MM centralization; G2 Control group: only OWHTO.

Abbreviations: ACL, anterior cruciate ligament; BPTB, bone‐patellar tendon‐bone; BMI, body mass index; F, females; LM, lateral meniscus; M, males; N, number; OWHTO, open‐wedge high tibial osteotomy; SD, standard deviation; /, not reported/not mentioned in the paper.

^a^ Subdivision of GA and GB is not reported.

^b^ Expressed as median with minimum and maximum.

^c^ Expressed as a percentage of Group 1 or Group 2.

### Surgical techniques and complications

All studies used arthroscopic meniscal centralization techniques with suture anchors, eventually combined with other procedures such as high tibial osteotomy (HTO), repair of the MMPRT, treatment of osteochondral lesions or ACL reconstruction. Complication rates were generally low; however, one study reported a 26.9% failure rate, primarily related to incomplete healing observed on MRI and subsequent progression to OA. Reintervention for complications included arthroplasty replacement in two cases. Surgical information, such as the centralization technique, the associated procedures and the complications, are summed up in Table 3.

**Table 3:** Surgical details: Centralization technique, associated procedures and complications.

Authors and publication year	Centralization technique	Associated injuries and/or treatments	Failures, *N* (%)	Type of complication/failures	Re‐intervention for complications	Additional surgery at distant follow‐up months^a^
Koga et al., 2016 [7]	Arthroscopic centralization with suture anchors; midlateral portal	GA: 5 patients double‐bundle ACL reconstruction with autologous semitendinosus graft; 1 mosaicplasty; 1 meniscal cyst resection	GA: 1/21 (4.5)	GA: 1 patient (had concomitant centralization + ACL reconstruction) at 16 months recurrent LM extrusion: tearing of the sutures + new flap tear at the posterior part (This patient was excluded for final analysis of post‐op results)	Flap tear excision and re‐centralization	/
		GB: saucerization concomitantly with centralization				
Koga et al., 2020 [8]	Arthroscopic meniscoplasty by capsular advancement with suture anchor; midlateral portal	17 patients microfracture for osteochondral lesions >3A ICRS; 1 patient double‐bundle ACL reconstruction with autologous semitendinosus graft	0	0	0	0 (1 patient: continuous pain despite 2 mm increase of the lateral joint space and arthroscopic findings with regeneration of meniscus‐like tissue. DFO at 3 y of follow‐up)
Mochizuki et al., 2021 [16]	MM arthroscopic centralization with suture anchors as an augmentation of MMPRT repair	Pullout repair of MMPRT with transtibial repair	6/26 (4 + 1 + 2) (26.9)	– At MRI 4 (15.4), there were cases of incomplete healing, healing rate of 84.6% at minimum of 2 years of follow‐up – 1 case progression OA Grades 2–3 KL – 2 arthroplasty replacement	2 arthroplasty replacement Survival rate 92.3%	/
Katagiri et al. 2021 [5]	G1: MM arthroscopic centralization with suture anchors + OWHTO	G1: OWHTO + 3 MM suture, 8 MM partial resection, 3 MM root tear pullout repair	G1: 1/21 (4.8)	G1: superficial surgical site infection	/	/
	G2: no centralization, only OWHTO	G2: OWHTO + 1 MM suture, 4 MM partial resection, 0 MM root tear pullout repair	G2:/	G2:/	/	/

*Note*: GA: Extrusion group with extruded LM of 3 mm or more; GB: Discoid group, with primary surgery for discoid LM + centralization. G1 Centralization group: OWHTO + MM centralization; G2 Control group: only OWHTO.

Abbreviations: ACL, anterior cruciate ligament; DFO, distal femoral osteotomy; ICRS, International Cartilage Repair Society grading system; KL, Kellgren–Lawrence; LM, lateral meniscus; MM, medial meniscus; mm, millimetres; MMPRT, medial meniscus posterior root tear; MRI, magnetic resonance imaging; N, number; OA, osteoarthritis; OWHTO, open‐wedge high tibial osteotomy; y, years; /, not reported/not mentioned in the paper.

^a^ Not directly related to the re‐intervention for complications but as additional surgery.

**Figure 3:**
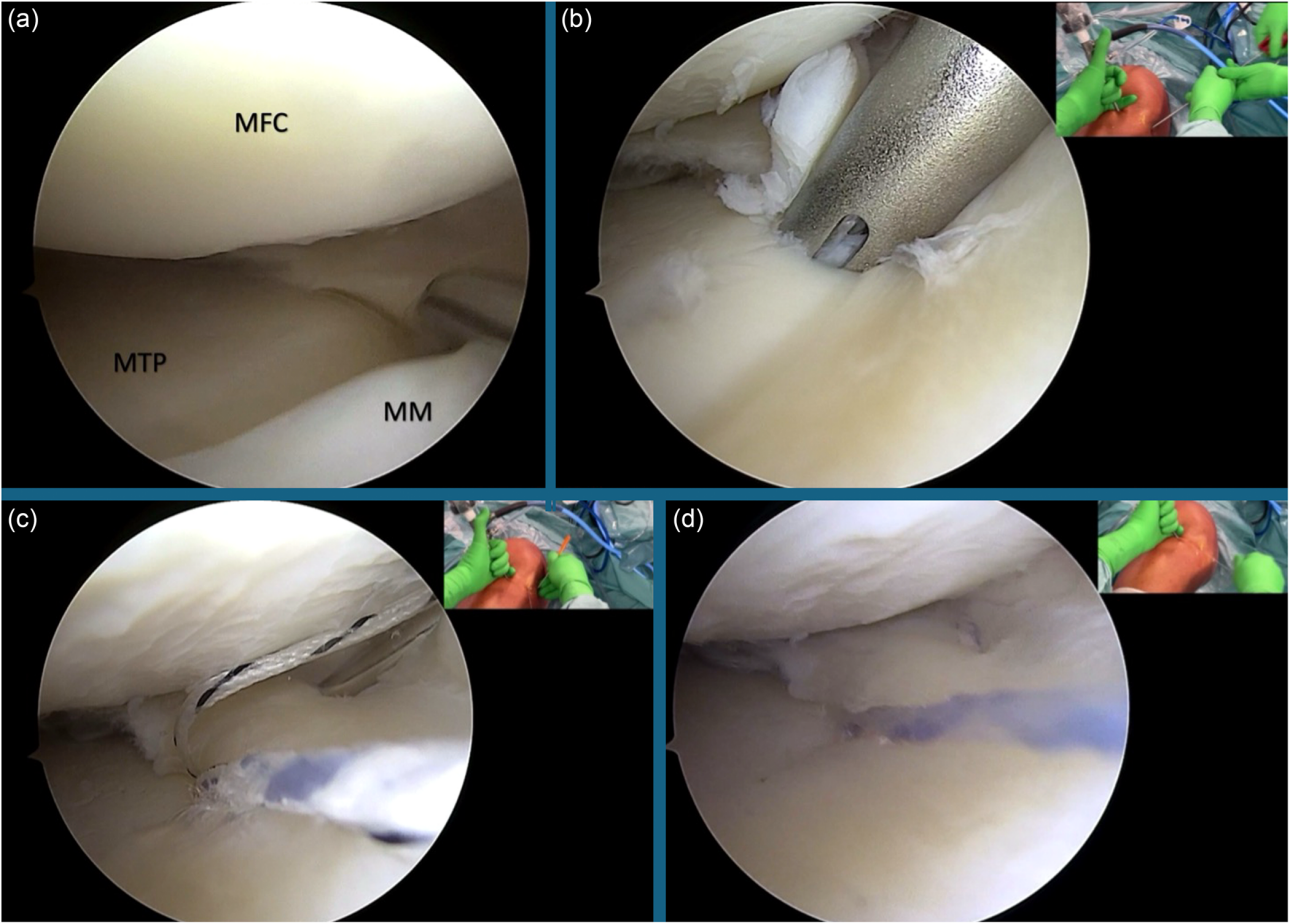
Surgical steps of a medial meniscus (MM) centralization with a suture anchor of a right knee. (a) A probe is used to assess the degree of medial meniscal extrusion. (b) The anchor is advanced through the medial supra‐meniscal portal with the spear into the bone. (c) The meniscus is traversed proximo‐distally and medio‐laterally with a suture passer (Microsuture Lasso, Arthrex Inc.) that is inserted via the medial supra‐meniscal portal. (d) The suture is retrieved from the anteromedial portal and is then pulled further until the desired repair tension is achieved. MFC, medial femoral condyle; MTP, medial tibial plateau.

An example of a suture anchor centralization technique is displayed in Figure 3.

### PROMs and clinical outcomes

Overall, meniscal centralization led to significant improvements in clinical outcomes.

For example, in Koga et al., the overall Lysholm score increased from 69.2 (SD: 14.5) to 96.5 (SD: 3.3; *p* < 0.0001), while KOOS pain improved from 72.2 (SD: 6.8) to 88.9 (SD: 9.8; *p* = 0.0010) and KOOS symptoms from 74.4 (SD: 9.7) to 91.1 (SD: 6.6; *p* = 0.0002). Their extruded meniscus subgroup (GA) showed slightly less improvement in KOOS pain (72.2–86.5, *p* = 0.056) compared to the discoid meniscus group (GB; 72.2–90.7, *p* < 0.0001). Similarly, Koga et al. reported significant post‐operative improvements in IKDC (51.8–75.8; *p* < 0.0001), Lysholm (64.9–92.8; *p* < 0.0001) and KOOS domains (pain: 64.9–88.5; symptoms: 61.4–81.6; both *p* < 0.0001). Mochizuki et al. observed significant increases in Lysholm (46.0 ± 8.8 to 78.5 ± 7.9; *p* < 0.05) and KOOS scores (pain: 47.4 ± 10.9 to 81.1 ± 6.1; symptoms: 53.2 ± 10.8 to 81.0 ± 9.7; both *p* < 0.05). Although Katagiri et al. did not report exact p‐values, mean Lysholm and KOOS scores improved similarly from the preoperative range (e.g., 71.8 for Lysholm) to higher post‐operative values (e.g., 94.1), further supporting the positive impact of meniscal centralization on knee function and symptom relief.

PROMs are detailed in Table 4, focusing on the IKDC, Lysholm score, and the pain and symptoms domains of the KOOS. Meanwhile, Table 5 presents additional KOOS domains, including sports and recreational function, quality of life and activities of daily living.

**Table 4:** Patient‐reported outcome measures—Part 1.

Authors and Publication year	IKDC			Lysholm score			KOOS pain			KOOS symptoms		
	Pre‐op	Post‐op	*p* value	Pre‐op	Post‐op	*p* value	Pre‐op	Post‐op	*p* value	Pre‐op	Post‐op	*p* value
Koga et al., 2016^a^ [7]	/	/	/	Overall: 69.2 (14.5)	Overall: 96.5 (3.3)	Overall: <0.0001	Overall: 72.2 (6.8)	Overall: 88.9 (9.8)	Overall: 0.0010	Overall: 74.4 (9.7)	Overall: 91.1 (6.6)	Overall: 0.0002
				GA: 70.4 (7.0)	GA: 95.6 (4.2)	GA: <0.0001	GA: 72.2 (7.6)	GA: 86.5 (13.5)	GA: 0.056	GA: 77.1 (7.8)	GA: 89.3 (7.4)	GA: 0.022
				GB: 68.4 (18.2)	GB: 97.1 (2.6)	GB: 0.0004	GB: 72.2 (3.9)	GB: 90.7 (5.9)	GB: <0.0001	GB: 72.5 (8.9)	GB: 92.4 (6.0)	GB: <0.0001
Koga et al., 2020^a^ [8]	51.8 (15.0)	75.8 (12.6)	<0.0001	64.9 (13.9)	92.8 (6.4)	<0.0001	64.9 (19.1)	88.5 (10.7)	<0.0001	61.4 (17.4)	81.6 (11.5)	<0.0001
Mochizuki et al., 2021^b^ [16]	/	/	/	46.0 ± 8.8 (25–57)	78.5 ± 7.9 (50–89)	<0.05	47.4 ± 10.9 (31–69)	81.1 ± 6.1 (64–100)	<0.05	53.2 ± 10.8 (25–71)	81.0 ± 9.7 (50–93)	<0.05
Katagiri et al. 2021^c^ [5]	/	G1: 71.8 (63.9–79.7)	/	/	G1: 94.1 (91.4–96.7)	/	/	G1: 82.9 (75.8–90.1)	/	/	G1: 81.8 (75.0–88.5)	/
	/	G2: 65.2 (57.5–73.0)	/	/	G2: 90.9 (86.0–95.8)	/	/	G2: 79.9 (70.6–89.1)	/	/	G2: 78.8 (69.6–88.0)	/

*Note*: Post‐op evaluation is at 2 years of follow‐up or at least 2 years of follow‐up. GA: Extrusion group with extruded LM of 3 mm or more; GB: Discoid group, with primary surgery for discoid lateral meniscus + centralization. G1 Centralization group: OWHTO + MM centralization; G2 Control group: only OWHTO.

Abbreviations: IKDC, International Knee Documentation Committee; KOOS, Knee injury and Osteoarthritis Outcome Score; OWHTO, open‐wedge high tibial osteotomy; post‐op, post‐operatively; pre‐op, preoperatively; SD, standard deviation; /, not reported/not mentioned in the paper.

^a^ Data are expressed as mean (SD).

^b^ Data are expressed as mean ± SD (range).

^c^ Data are expressed as mean with a 95% confidence interval.

**Table 5:** Patient‐reported outcome measures—Part 2.

Authors and publication year	KOOS sport and recreational function			KOOS quality of life			KOOS activities of daily living		
	Pre‐op	Post‐op	*p* value	Pre‐op	Post‐op	*p* value	Pre‐op	Post‐op	*p* value
Koga et al., 2016^a^ [7]	Overall: 41.7 (18.9)	Overall:78.8 (23.8)	Overall: 0.0028	Overall: 45.8 (25.8)	Overall: 78.1 (17.5)	Overall: 0.0029	Overall: 88.5 (5.7)	Overall: 94.4 (7.4)	Overall: 0.091
	GA: 45.0 (19.0)	GA: 72.1 (32.6)	GA: 0.13	GA: 43.8 (28.3)	GA: 75.0 (21.3)	GA: 0.053	GA: 88.8 (6.2)	GA: 91.2 (10.3)	GA: 0.66
	GB: 39.3 (12.1)	GB: 83.3 (14.6)	GB: <0.0001	GB: 47.3 (14.8)	GB: 80.6 (14.8)	GB: 0.0005	GB: 88.4 (3.1)	GB: 96.6 (2.8)	GB: <0.0001
Koga et al., 2020^a^ [8]	38.7 (24.8)	72.9 (20.3)	<0.0001	36.5 (20.7)	69.7 (19.3)	<0.0001	79.1 (17.5)	95.5 (4.5)	<0.0001
Mochizuki et al., 2021^b^ [16]	18.9 ± 6.1 (5–35)	66.4 ± 5.8 (55–75)	<0.05	34.9 ± 11.4 (12–50)	68.2 ± 13.6 (38–88)	<0.05	64.6 ± 10.5 (49–75)	86.3 ± 6.6 (66–100)	<0.05
Katagiri et al. 2021^c^ [5]	/	G1: 67.1 (56.0–78.3)	/	/	G1: 67.9 (56.6–79.1)	/	/	G1: 90.3 (84.9–95.7)	/
	/	G2: 63.3 (47.7–78.9)	/	/	G2: 68.1 (56.5–79.7)	/	/	G2: 90.8 (86.0–95.6)	/

*Note*: Post‐op evaluation is at 2 years of follow‐up or at least 2 years of follow‐up. GA: Extrusion group with extruded LM of 3 mm or more; GB: Discoid group, with primary surgery for discoid lateral meniscus + centralization. G1 Centralization group: OWHTO + MM centralization; G2 Control group: only OWHTO.

Abbreviations: KOOS, Knee injury and Osteoarthritis Outcome Score; OWHTO, open‐wedge high tibial osteotomy; post‐op, post‐operatively; pre‐op, preoperatively; SD, standard deviation; /, not reported/not mentioned in the paper.

^a^ Data are expressed as mean (SD).

^b^ Data are expressed as mean ± SD (range).

^c^ Data are expressed as mean with a 95% confidence interval.

Range of motion (ROM) improved post‐operatively, although not all changes were statistically significant. Koga et al. [7] reported a slight improvement in extension from 1.5° to 0.1° (*p* = 0.057) and flexion from 4.0° to 0.5° (*p* = 0.10). Katagiri et al. [5] showed small, non‐significant improvements in both extension and flexion angles. Table 6 summarizes the clinical evaluations, including the McMurray test results, subjective recovery and patient satisfaction and ROM measurements.

**Table 6:** Clinical evaluation and knee angles.

Authors and publication year	Clinical evaluation						Range of motion					
	McMurray positive[Link], a			Subjective recovery/patient satisfaction			Extension^b^			Flexion^b^		
	Pre‐op	Post‐op	*p* value	Pre‐op	Post‐op	*p* value	Pre‐op	Post‐op	*p* value	Pre‐op	Post‐op	*p* value
Koga et al., 2016^c^ [7]	Overall: 17	Overall: 1 (only click not pain)	<0.0001	Overall: 21.6 (20.9)^d^	Overall: 84.2 (19.4)^d^	Overall: <0.0001^d^	Overall: 1.5 (3.1)^e^	Overall: 0.1 (0.3)^e^	Overall: 0.057^e^	Overall: 4.0 (10.1)^e^	Overall: 0.5 (1.5)^e^	Overall: 0.10^e^
				GA: 27.5 (16.7)^d^	GA: 82.5 (19.8)^d^	GA: <0.0001^d^						
				GB: 17.7 (22.2)^d^	GB: 85.3 (12.1)^d^	GB: <0.0001^d^						
Koga et al., 2020^c^ [8]	– Positive for pain: 21 – Positive for a click: 12	– Positive for pain: 2 – Positive for a click: 4	– Pain <0.0001 – Click 0.0352	32.4 (23.8)^d^	79.9 (12.1)^d^	<0.0001^d^	1.9 (3.1)^e^	2.2 (3.8)^e^	0.1780^e^	4.3 (8.2)^e^	0.1 (0.4)^e^	0.2162^e^
Mochizuki et al., 2021 [16]	/	/	/	/	/	/	/	/	/	/	/	/
Katagiri et al. 2021^f^ [5]	/	/	/	G1: 20 (0–70)^g^	G1: ≈70^g^^,^ h	/	G1: −2 (−10 to 3)	G1: −1 (−3 to 2)	0.21	G1: 140 (130–155)	G1: 145 (125–155)	0.23
	/	/	/	G2: 50 (0–80)^g^	G2: ≈70^g^^,^ h	/	G2: 0 (−10 to 5)	G2: 0 (−3 to 5)	0.50	G2: 140 (100–150)	G2: 145 (125–155)	0.06

*Note*: GA: Extrusion group with extruded LM of 3 mm or more; GB: Discoid group, with primary surgery for discoid lateral meniscus + centralization. G1 Centralization group: OWHTO + MM centralization; G2 Control group: only OWHTO.

Abbreviations: OWHTO, open‐wedge high tibial osteotomy; Post‐op, post‐operatively; Pre‐op, preoperatively; SD, standard deviation; /, not reported/not mentioned in the paper.

^a^ Positive for a click and/or pain, otherwise specified.

^b^ Expressed in degrees.

^c^ Values expressed in mean (SD).

^d^ Post‐op patient satisfaction measured on a 100 mm visual analogue scale in 1 mm increments. Post‐op considered at 2 years of follow‐up, otherwise specified.

^e^ Side‐to‐side difference between injured and uninjured legs.

^f^ Valued expressed as median with minimum and maximum.

^g^ Patient subjective satisfaction evaluated out of 100 points score on the response to questionnaires to 3 years after surgery.

^h^ Interpreted from plot iconography, not reported in precise numbers.

#### Radiographic results

Radiographic assessments showed improvements in joint space width (JSW) and hip–knee–ankle (HKA) angles post‐operatively. MRI evaluations showed a significant reduction in meniscal extrusion, with one study reporting a reduction from 5.0 mm preoperatively to 1.0 mm post‐operatively (*p* < 0.0001). X‐ray evaluations and MRI findings are presented in Table 7 and Table 8, respectively.

**Table 7:** X‐ray imaging evaluation. Post‐op evaluation is at 2 years of follow‐up.

Authors and publication year	X‐rays evaluation														
	Lateral JSW^a^ (mm)			Lateral JSW (mm)^b^			HKA angle (degrees)			Medial JSW (mm)^c^,^d^			JLCA (degrees)^d^^,^ e		
	Pre‐op	Post‐op	*p* value	Pre‐op	Post‐op	*p* value	Pre‐op	Post‐op	*p* value	Pre‐op	Post‐op	*p* value	Pre‐op	Post‐op	*p* value
Koga et al., 2016 [7]	GA: 4.8 mm (3–6)^f^	GA: 5.6 mm (4–7)^f^	GA: 0.041	/	/	/	/	/	/	/	/	/			
	GB: 5.4 mm (3–8)^f^	GB: 5.5 mm (3–8)^f^	GB:/												
Koga et al., 2020 [8]	2.1 mm (0–4)^f^	3.3 mm (0–6)^f^	0.0001	3.3 mm (0–5)^f^	4.7 mm (2–6)^f^	<0.0001	/	/	/	/	/	/	/	/	/
Mochizuki et al., 2021 [16]	/	/	/	/	/	/	Valgus −2.4 ± 1.0 (−3.9 to 0)^g^	Valgus −2.2 ± 1.2 (−3.8 to 1.6)^g^	0.13	/	/	/	/	/	/
Katagiri et al., 2021 [5]	/	/	/	/	/	/	G1: 8.5 (2.7–11.8)^h^	G1: −1.6 (−5.1 to 3.0)^h^	/	G1: 1.9 (1.4–2.4)	G1: 2.7 (2.3–3.2)	G1: <0.01	G1: 4.2 (3.3–5.1)	G1: 3.3 (2.6–3.9)	0.03
	/	/	/	/	/	/	G2: 7.7 (3.6–13.6)^h^	G2: −0.3 (−6.4 to 5.0)^h^	/	G2: 2.2 (1.5–2.9)	G2: 2.2 (1.5–3.0)	0.83	G2: 3.9 (2.9–4.9)	G2: 3.5 (2.1–4.9)	0.17

*Note*: GA: Extrusion group with extruded LM of 3 mm or more; GB: Discoid group, with primary surgery for discoid lateral meniscus + centralization. G1 Centralization group: OWHTO + MM centralization; G2 Control group: only OWHTO. HKA angle: mechanical alignment evaluated by HKA angle defined as an angle between the axis from the centre of the femoral head to the inter‐condylar notch and the axis from the tibial spine to the centre of the talus on a plain full weight bearing x‐rays.

Abbreviations: HKA, hip–knee–ankle; JLCA, joint line convergence angle; JSW, joint space width; mm, millimetres; OWHTO, open‐wedge high tibial osteotomy; Post‐op, post‐operatively; Pre‐op, preoperatively; SD, standard deviation; /, not reported/not mentioned in the paper.

^a^ Measured on standing 45° flexion postero‐anterior view. The absolute JSW was measured at the centre of the lateral compartment in 1‐mm increments.

^b^ Measured on conventional standing extension anteroposterior view.

^c^ JSW measured at the narrowest point in the medial compartment.

^d^ Values are expressed as mean with a 95% confidence interval.

^e^ JLCA measured as the angle between joint orientation lines at the distal femur and the proximal tibia.

^f^ Values are expressed as mean (range).

^g^ Values are expressed as mean ± SD (range).

^h^ Values are expressed as median with minimum and maximum.

**Table 8:** MRI evaluation.

Authors and publication year	MRI evaluation											
	MEW^a^ (mm)			Full length of MM (mm)^b^			Extrusion distance of MM (mm)^b^			MME ratio (%)^b^^,^ c		
	Pre‐op	Post‐op^d^	*p* value	Pre‐op	Post‐op^e^	*p* value	Pre‐op	Post‐op^e^	*p* value	Pre‐op	Post‐op^e^	*p* value
Koga et al., 2016 [7]	GA: 5.0 mm (3–9)^f^	GA: 1.0 mm (0–3)^f^	GA: <0.0001	/	/	/	/	/	/	/	/	/
	GB: 1.6 mm (0–5)^f^	GB: 0.3 mm (0–3)^f^	GB: 0.047									
Koga et al., 2020 [8]	4.9 mm (3–8)^f^	1.1 mm (0–3)^f^	0.0006	/	/	/	/	/	/	/	/	/
Mochizuki et al., 2021 [16]	/	/	/	11.9 ± 1.1 (9.6–14.2)	12.1 ± 1.4 (9.7–15.7)	0.08	4.8 ± 0.7 (3.4–6.5)	2.7 ± 0.3 (2.1–3.2)	<0.05	40.2 ± 7.0 (28.7–57.0)	22.6 ± 3.6 (16.5–32.0)	<0.05
Katagiri et al., 2021 [5]	/	/	/	/	/	/	/	/	/	/	/	/
	/	/	/	/	/	/	/	/	/	/	/	/

*Note*: GA: Extrusion group with extruded LM of 3 mm or more; GB: Discoid group, with primary surgery for discoid lateral meniscus + centralization.

Abbreviations: MEW, meniscal extrusion width; MM, medial meniscus; mm, millimetres; MME, medial meniscus extrusion; MRI, magnetic resonance imaging; Post‐op, post‐operatively; Pre‐op, preoperatively; SD, standard deviation; /, not reported/not mentioned in the paper.

^a^ MRI examination with the patient supine with the knee fully extended, MEW considered as the distance from the most peripheral aspect of the meniscus to the border of the tibia, excluding any osteophytes, measured in 1‐mm increments.

^b^ Values are expressed as mean ± SD (range).

^c^ MME ratio defined as the extrusion distance divided by the MM full length.

^d^ Post‐op evaluation is at 1 year of follow‐up.

^e^ Post‐op evaluation is at 2 years of follow‐up.

^f^ Values are expressed as mean (range).

#### Post‐operative protocols and return to sports

While all studies emphasized early ROM and a gradual return to activity, there were differences in weight‐bearing times and specific protocols.

All studies implemented structured rehabilitation protocols that began with early ROM exercises, with full return to activity typically occurring at 4–6 months post‐operatively. Since the four included articles used different post‐operative protocols, they are presented in Table 9, along with the return to sports.

**Table 9:** Post‐operative protocols and return to sport.

Authors and publication year	Post‐operative protocol^a^	Return to sport (sport performance level^b^^,^ c)		
		Pre‐op	Post‐op	*p* value
Koga et al., 2016 [7]	Immediately ROM exercises without restriction. Full weight bearing with a knee immobilizer was allowed for the first 4 weeks. After 4 weeks, full weight bearing without the knee immobilizer. Running at 2.5 months with progressive squatting exercises, and deep squatting was allowed after 3 months. Patients progressed to full activity after 4 months.	Overall: 14.6 (19.0)	Overall: 82.2 (23.4)	Overall: <0.0001
		GA: 16.7 (8.2)	GA: 78.3 (29.9)	GA: 0.0007
		GB: 13.3 (19.1)	GB: 84.8 (16.7)	GB: <0.0001
Koga et al., 2020 [8]	Immediately ROM exercises without restriction. Partial weight bearing with a knee immobilizer for the first 4 weeks. After 4 weeks, partial weight bearing without the knee immobilizer, with progression to full weight bearing at 6 weeks. Deep squatting after 3 months; running was allowed at 3 months; patients progressed to full activity after 6 months.	38.7 (28.7)	72.6 (14.9)	<0.0001
Mochizuki et al., 2021 [16]	A knee brace locked at 20° flexion was used for the first week, and ROM exercises started 1 week after surgery. Non‐weight bearing for the first 2 weeks. A 33% partial weight bearing from 2 weeks after surgery, and full weight bearing at 4 weeks after surgery. Jog at 4 months after surgery. At 6 months, full activity and sports were allowed.	/	/	/
Katagiri et al. 2021 [5]	The post‐operative rehabilitation did not vary with the meniscal procedure between G1 and G2. ROM and quadriceps setting exercises are performed the day after surgery. One third, two thirds and full weight bearing were initiated 3, 10 and 14 days after surgery. Running exercises were started at 3 months after confirming bone union and progressed to full activity after 6 months post‐operatively.	/	/	/

*Note*: GA: Extrusion group with extruded LM of 3 mm or more; GB: Discoid group, with primary surgery for discoid lateral meniscus + centralization. G1 Centralization group: OWHTO + MM centralization; G2 Control group: only OWHTO.

Abbreviations: mm, millimetres; OWHTO, open‐wedge high tibial osteotomy; Post‐op, post‐operatively; Pre‐OP, preoperatively; ROM, range of motion; SD, standard deviation; /, not reported/not mentioned in the paper.

^a^ To consider always the associated procedures.

^b^ Measured on a 100‐mm visual analogue scale in 1 mm increments.

^c^ Data are represented as mean (SD). Post‐op is considered at 2 years of follow‐up.

Return to sports was a common outcome measure, with most studies reporting significant improvements in sports participation. Post‐operative protocols varied slightly but generally allowed for return to full activity and sports within 6 months.

## DISCUSSION

The main finding of this systematic review was that meniscal centralization significantly improves clinical outcomes and reduces meniscal extrusion with minimal complications in patients with symptomatic meniscal extrusion. These results highlight the potential of meniscal centralization as an effective treatment modality for patients with knee pain and loss of function related to meniscal extrusion.

In a recent study aiming to describe the characteristics of isolated meniscal extrusion, its imaging features, and clinical correlations, the authors found that 68% of patients were symptomatic, with knee pain correlating with the side of extrusion [10]. MT ligament abnormalities were present in 65% of cases, and patients with ≥3 mm of extrusion were significantly more likely to have these abnormalities (100%) than those with less than 3 mm (36%). Also, an association was found between MT ligament loosening and meniscal extrusion in patients with symptomatic discoid lateral menisci [19]. This evidence suggests that there may be an opportunity to address MT ligament abnormalities with meniscal centralization techniques in cases of isolated meniscal extrusion, particularly with ≥3 mm of extrusion. Also, meniscal centralization may be a viable early intervention before the progression to an MMPRT [11]. Krych et al. conducted a study evaluating the relationship between the MT ligament, medial meniscal extrusion, and MMPRTs, as well as the progression of meniscal extrusion over time [11]. A total of 27 knees from 26 symptomatic patients were included, and 63 MRI scans were analyzed. Results showed that all patients had MT ligament rupture and medial meniscal extrusion prior to the development of MMPRTs, with mean extrusion increasing from 3.3 to 5.5 mm over a mean of 1.7 years. These findings suggest that MT ligament rupture and meniscal extrusion are early events that predispose patients to MMPRTs and provide a possible explanation for why meniscal extrusion often persists despite medial meniscal root repair. So, meniscal extrusion seems to be the *primum movens* that leads to knee pathology, including but not limited to OA progression [13].

In a large cohort study of 253 patients with subchondral insufficiency fractures of the knee (SIFK), a strong association was found with meniscal extrusion ≥3.0 mm [2]. The study found that 77.1% of patients had medial meniscal tears, of which 91% had significant meniscal extrusion. SIFK was more common in varus knees and predominantly involved the medial femoral condyle and medial tibial plateau, suggesting also a role in the lower limb axis in the development of this condition.

Meniscus centralization is a relatively recent practice, first introduced by Koga et al. in 2012 through a technical note [9]. Since then, various techniques have been developed, but there remains a paucity of long‐term clinical outcome data and survivorship [18]. In this review, the follow‐up period ranged from 24 to 35 months, with a minimum follow‐up of 24 months. In this timeframe, PROMs demonstrated a significant improvement across all the studies, and the complication rates were generally low, with the exception of one study that reported a 26.9% failure rate, primarily due to incomplete healing observed on MRI. The current review found that there was a significant reduction in meniscal extrusion post‐operatively, as well as improvements in JSW at post‐operative radiographic assessment, showing the effectiveness of meniscal centralization in reducing extrusion and consequently potentially reducing the risk of developing significant knee pathologies, such as MMPRTs, SIFK and OA.

The results of this review suggest that meniscal centralization may improve patient‐reported outcomes by reducing meniscal extrusion and mitigating its negative impact on joint biomechanics. Although the evidence base is limited, surgeons may consider this procedure in patients with symptomatic extrusion. Early intervention, possibly combined with additional procedures such as root repair or corrective osteotomies if needed, may improve long‐term joint preservation.

### Limitations

This systematic review is limited by the heterogeneity of the included studies, particularly in terms of patient populations, surgical techniques and post‐operative protocols.

The limited number of studies included, their different patient populations, surgical techniques and post‐operative protocols significantly limit the interpretability of our findings. This heterogeneity prevents a robust data synthesis and may introduce bias or confounding factors that are difficult to isolate. In addition, the small sample sizes in each study limit statistical power and reduce confidence in generalizing the results to larger patient groups.

The different follow‐up periods and the retrospective nature of some studies may also affect the generalizability of the findings. Despite these limitations, the review provides a comprehensive analysis of the current evidence supporting the use of meniscal centralization for symptomatic meniscal extrusion.

Future research should prioritize larger, well‐designed comparative studies—possibly randomized controlled trials—to better define the role of meniscal centralization, refine surgical indications and provide clearer guidance on optimal post‐operative management.

## CONCLUSIONS

The systematic review indicates that meniscal centralization techniques are effective in improving clinical outcomes in patients with symptomatic meniscal extrusion. Significant improvements in clinical scores, reduction in meniscal extrusion, and knee function observed across studies support these surgical procedures. Also, minimal complications further suggest that these procedures are safe and beneficial for the treatment of meniscal extrusion. However, although early clinical outcomes are promising, future comparative studies with well‐defined indications are necessary to confirm the efficacy of the centralization procedure.

## AUTHOR CONTRIBUTIONS

Alessandro Carrozzo and Francesco Bosco have contributed substantially to conception and design, data acquisition, analysis, and interpretation. They agree to be accountable for all aspects of the work in ensuring that questions related to the accuracy or integrity of any part of the work are appropriately investigated and resolved. Leandro Ramazzini, Fortunato Giustra and Virginia Masoni have contributed substantially to the data analysis, interpretation and manuscript drafting. Marcello Capella, Michele Malavolta, Jae‐Sung An and Hideyuki Koga have significantly contributed to revising the manuscript critically for important intellectual content, giving final approval of the version to be published.

## CONFLICT OF INTEREST STATEMENT

Michele Malavolta is consultant for Medacta. Hideyuki Koga is Speaker for Arthrex, Taisho Pharmaceutical Holdings Paid Consultant for Olympus Terumo Biomaterials. Support received from Smith & Nephew, Zimmer Biomet, Stryker, Olympus Terumo Biomaterials. Editorial or Governing board of *Journal of Orthopaedic Science*, *Annals of Joint*, *Asia‐Pacific Journal of Sports Medicine, Arthroscopy, Rehabilitation and Technology* and *Knee Surgery & Related Research*. The remaining authors declare no conflicts of interest.

## ETHICS STATEMENT

The ethics statement is not available.

## Data Availability

The data set analyzed in this study is available from the corresponding author on reasonable request.

## References

[1] Allaire R , Muriuki M , Gilbertson L , Harner CD . Biomechanical consequences of a tear of the posterior root of the medial meniscus. Similar to total meniscectomy. J Bone Joint Surg Am. 2008;90(9):1922–1931. 18762653 10.2106/JBJS.G.00748

[2] Clark SC , Pareek A , Hevesi M , Okoroha KR , Saris DBF , Camp CL , et al. High incidence of medial meniscus root/radial tears and extrusion in 253 patients with subchondral insufficiency fractures of the knee. Knee Surg Sports Traumatol Arthrosc. 2024;32:2755–2761. 38769782 10.1002/ksa.12271

[3] Daney BT , Aman ZS , Krob JJ , Storaci HW , Brady AW , Nakama G , et al. Utilization of transtibial centralization suture best minimizes extrusion and restores tibiofemoral contact mechanics for anatomic medial meniscal root repairs in a cadaveric model. Am J Sports Med. 2019;47(7):1591–1600. 31091129 10.1177/0363546519844250

[4] Gilat R , Mitchnik IY , Mimouni T , Agar G , Lindner D , Beer Y . The meniscotibial ligament role in meniscal extrusion: a systematic review and meta‐analysis. Arch Orthop Trauma Surg. 2023;143(9):5777–5786. 37266692 10.1007/s00402-023-04934-7

[5] Katagiri H , Nakagawa Y , Miyatake K , Ohara T , Shioda M , Sekiya I , et al. Short‐term outcomes after high tibial osteotomy aimed at neutral alignment combined with arthroscopic centralization of medial meniscus in osteoarthritis patients. J Knee Surg. 2023;36(3):261–268. 34261157 10.1055/s-0041-1731738

[6] Keyhani S , Movahedinia M , LaPrade RF , Qoreishy M , Vosoughi F . Long‐term clinical results of using a posteromedial all‐inside and anteromedial inside‐out approach to repair unstable or irreducible bucket‐handle medial meniscal tears. J Orthop Traumatol. 2023;24(1):12. 37024629 10.1186/s10195-023-00691-w PMC10079791

[7] Koga H , Muneta T , Watanabe T , Mochizuki T , Horie M , Nakamura T , et al. Two‐year outcomes after arthroscopic lateral meniscus centralization. Arthroscopy. 2016;32(10):2000–2008. 27132775 10.1016/j.arthro.2016.01.052

[8] Koga H , Nakamura T , Katagiri H , Nakagawa Y , Ozeki N , Ohara T , et al. Two‐year outcomes after meniscoplasty by capsular advancement with the application of arthroscopic centralization technique for lateral compartment knee osteoarthritis. Am J Sports Med. 2020;48(13):3154–3162. 33026837 10.1177/0363546520957367

[9] Koga H , Nakamura T , Nakagawa Y , Ozeki N , Ohara T , Shioda M , et al. Arthroscopic centralization using knotless anchors for extruded medial meniscus. Arthrosc Tech. 2021;10(3):e639–e645. 33738196 10.1016/j.eats.2020.10.051 PMC7953036

[10] Krych AJ , Bernard CD , Leland DP , Camp CL , Johnson AC , Finnoff JT , et al. Isolated meniscus extrusion associated with meniscotibial ligament abnormality. Knee Surg Sports Traumatol Arthrosc. 2020;28(11):3599–3605. 31332493 10.1007/s00167-019-05612-1

[11] Krych AJ , LaPrade MD , Hevesi M , Rhodes NG , Johnson AC , Camp CL , et al. Investigating the chronology of meniscus root tears: do medial meniscus posterior root tears cause extrusion or the other way around? Orthop J Sports Med. 2020;8(11):2325967120961368. 33209944 10.1177/2325967120961368 PMC7645763

[12] Krych AJ , Reardon PJ , Johnson NR , Mohan R , Peter L , Levy BA , et al. Non‐operative management of medial meniscus posterior horn root tears is associated with worsening arthritis and poor clinical outcome at 5‐year follow‐up. Knee Surg Sports Traumatol Arthrosc. 2017;25(2):383–389. 27761625 10.1007/s00167-016-4359-8

[13] Langhans MT , Lamba A , Saris DBF , Smith P , Krych AJ . Meniscal extrusion: diagnosis, etiology, and treatment options. Curr Rev Musculoskelet Med. 2023;16(7):316–327. 37191818 10.1007/s12178-023-09840-4 PMC10356705

[14] LaPrade RF , Matheny LM , Moulton SG , James EW , Dean CS . Posterior meniscal root repairs: outcomes of an anatomic transtibial pull‐out technique. Am J Sports Med. 2017;45(4):884–891. 27919916 10.1177/0363546516673996

[15] Mariani PP , Torre G , Battaglia MJ . The post‐traumatic meniscal extrusion, sign of meniscotibial ligament injury. A case series. Orthop Traumatol Surg Res. 2022;108(3):103226. 35123034 10.1016/j.otsr.2022.103226

[16] Mochizuki Y , Kawahara K , Samejima Y , Kaneko T , Ikegami H , Musha Y . Short‐term results and surgical technique of arthroscopic centralization as an augmentation for medial meniscus extrusion caused by medial meniscus posterior root tear. Eur J Orthop Surg Traumatol. 2021;31(6):1235–1241. 33475853 10.1007/s00590-021-02874-9

[17] Nakamura T , Koga H . Review of the development of meniscus centralization. Curr Rev Musculoskelet Med. 2024;17(8):303–312. 38760631 10.1007/s12178-024-09905-y PMC11219636

[18] Nishino K , Hashimoto Y , Iida K , Kinoshita T , Nakamura H . Intrameniscal degeneration and meniscotibial ligament loosening are associated factors with meniscal extrusion of symptomatic discoid lateral meniscus. Knee Surg Sports Traumatol Arthrosc. 2023;31(6):2358–2365. 36112159 10.1007/s00167-022-07161-6

[19] Paletta GA , Crane DM , Konicek J , Piepenbrink M , Higgins LD , Milner JD , et al. Surgical treatment of meniscal extrusion: a biomechanical study on the role of the medial meniscotibial ligaments with early clinical validation. Orthop J Sports Med. 2020;8(7):2325967120936672. 32775474 10.1177/2325967120936672 PMC7391441

[20] Wu TY . Arthroscopic medial meniscus posterior root repair with centralization using knotless suture anchors. Arthrosc Tech. 2022;11(4):e661–e668. 35493038 10.1016/j.eats.2021.12.019 PMC9052078

